# Preconceptional Immunization Can Modulate Offspring Intrathymic IL-17-Producing γδT Cells with Epigenetic Implications Mediated by microRNAs

**DOI:** 10.3390/ijms22126633

**Published:** 2021-06-21

**Authors:** Thamires Rodrigues de-Sousa, Rodrigo Pessôa, Andrezza Nascimento, Beatriz Oliveira Fagundes, Fábio da Ressureição Sgnotto, Alberto José da Silva Duarte, Sabri Saeed Sanabani, Jefferson Russo Victor

**Affiliations:** 1 Laboratory of Medical Investigation LIM-56, Division of Clinical Dermatology, Medical School, University of São Paulo, São Paulo 05403-000, Brazil; thamires@gmail.com (T.R.d.-S.); rodrigo_pessoa1@hotmail.com (R.P.); andrezza.ns@gmail.com (A.N.); bibifags73@gmail.com (B.O.F.); 2 Division of Hematology, Medical School, University of São Paulo, São Paulo 01246-000, Brazil; fabio.house@hotmail.com; 3 Division of Pathology, Medical School, University of São Paulo, São Paulo 05403-000, Brazil; adjsduar@usp.br; 4 Faculdades Metropolitanas Unidas (FMU), School of Health Sciences, São Paulo 04505-002, Brazil; 5 Medical School, Santo Amaro University (UNISA), São Paulo 04829-300, Brazil

**Keywords:** mice, thymus, gamma-delta T cells, IL-17, microRNA

## Abstract

The mechanisms through which maternal immunization can modulate offspring thymic maturation of lymphocytes are not fully understood. Here, we aimed to evaluate whether maternal OVA-immunization can inhibit the maturation of IL-17-producing γδT cells in offspring thymus, and if this mechanism has epigenetic implications mediated by microRNAs (miRNAs) expression. Wild-type (WT) C57BL/6 females were immunized with OVA in Alum or Alum alone and were mated with normal WT males. Evaluating their offspring thymus at 3 or 20 days old (d.o.), we observed that maternal OVA immunization could inhibit the thymic frequency of offspring CD27- and IL-17^+^ γδT cells at the neonatal and until 20 days old. Furthermore, we evaluated the expression of function-related γ and δ variable γδTCR chains (Vγ1, Vγ2, Vγ3, Vδ4, and Vδ6.3), observing that maternal OVA-immunization inhibits Vγ2 chains expression. The small RNAs (sRNAs), particularly miRNAs, and messenger RNAs (mRNA) expression profiles by pools of thymus tissue samples (from 9 to 11 mice) from offspring OVA-immunized or Alum-immunized mothers were analyzed via Illumina sequencing platform and bioinformatics approaches. Using a fold change >4, our results showed that seven miRNAs (mmu-miR-126a-3p, 101a-3p, 744-3p,142-5p, 15a-5p, 532-5p, and 98-5p) were differentially expressed between both groups. Ten target genes were predicted to interact with the seven selected miRNAs. There were no enriched categories of gene ontology functional annotation and pathway enrichment analysis for the target genes. Interestingly, four of the identified miRNAs (mmu-miR-15a, mmu-miR-101 mmu-miR-126, and mmu-miR-142) are related to IL-17 production. Our data is of significance because we demonstrate that maternal immunization can modulate offspring thymic maturation of IL-17-producing γδT cells possibly by an epigenetic mechanism mediated by miRNAs.

## 1. Introduction

Several groups evidenced the regulation of offspring allergy development mediated by maternal immunization with allergens. This mechanism depends on the timing and intensity of prenatal and postnatal allergen exposure of the mother [1]. However, it seems closely related to preconception immunization, as evidenced in murine models with the allergen OVA [2,3,4,5] and the dust mite Dermatophagoides pteronyssinus [6,7]. Mainly, this influence can be related to passive transference of maternal anti-allergen IgG and the peripheral inhibition of offspring Th2 related cytokines and IgE production [8,9,10,11]. Nevertheless, when analyzing the influence of maternal IgG on an offspring primary organ as the thymus, it could be observed that the maternal influence can affect Th17 cells without influence on Th1 and Th2 cells [12] and, due to biological similarities, can also involve the maturation of IL-17-producing γδ T [13]. The involvement of IL-17 producing γδT cells have been discussed in the main clinical manifestations of allergic diseases [14,15,16], and the modulatory effect of murine maternal allergen immunization on offspring thymic IL-17 producing γδT cells thymus was suggested to mediate this effect in mouse and human [17].

Some hypotheses discussed that maternal immunization with allergens could broadly modulate offspring intrathymic maturation of T cells [18,19,20] and had been experimentally evidenced for murine and human γδT, Treg and Breg cells [12,21,22,23,24,25,26,27,28]. However, no molecular pieces of evidence have been obtained to elucidate if this mechanism involves some epigenetic alterations on modulated cells.

The production of IFN-γ or IL-17 cytokines, yields the placing of two principal subsets of murine γδT cells [29]. Phenotypically, these subsets can be identified by the expression of CD27 molecule that is expressed only by IFN-γ-producing γδT cells; therefore, the CD27- phenotype is related to IL-17-producing γδT cells [29,30]. Some additional functional γδT cells properties, including the secretion of IFN-γ or IL-17 cytokines, can also be observed based on the function-related γ and δ variable γδTCR chains expression [31].

Regarding the Vγ chains expression, Vγ1^+^ γδT cells can be related lymphoid tissue homing and IFN-**γ** production [32], Vγ2^+^ γδT cells can be related to skin homing and a restriction to IL-17-production when compared Vγ1 expression [33,34,35,36], Vγ3^+^ γδT cells can be related to epidermal homing, but no cytokine-related functionality is described in the literature. Regarding Vδ chains expression, Vδ4^+^ γδT cells can be related to the production of IL-17 [37] and Vδ6.3^+^ γδT cells can be related to the production of IL-4 and IFN-γ [38].

Small RNAs, including miRNAs, are endogenous, ~22-nt Alum-coding RNAs (sncRNAs) that modulate the expression of target genes translation through binding with imperfect complementarity to the 3′UTR of their target mRNAs [39,40]. Few miRNAs have been characterized in the γδ T cell, including miR-133b and miR-206, which are co-regulated with IL-17 but have no effect on the production cytokine [41]. Besides, a recent study by Schmolka and colleagues [42] detected significant expression of miR-146a in γδ27- T cells compared with their γδ27^+^ T cell counterparts and revealed that miR-146a inhibits the γδ T cells to produce the IFN-γ and thus limits the γδ27− T cells functional plasticity by targeting Nod1 in vitro and in vivo.

While the impact of maternal immunization on IL-17 producing γδT cells maturation seems to be implicated with the development of murine and human allergies, no epigenetic implications were evaluated. Therefore, this study aimed to elucidate this issue in a standardized murine protocol of offspring allergy inhibition mediated by maternal allergen immunization.

In this study, we aimed to determine whether maternal immunization on IL-17-producing γδT cells maturation is implicated with the development of murine allergies and that miRNAs are involved in regulating this process.

## 2. Results

### 2.1. Maternal Immunization Could Inhibit Offspring Allergy and Reduce the Frequency of Offspring Thymic IL-17-Producing γδT Cells

**Figure 1:**
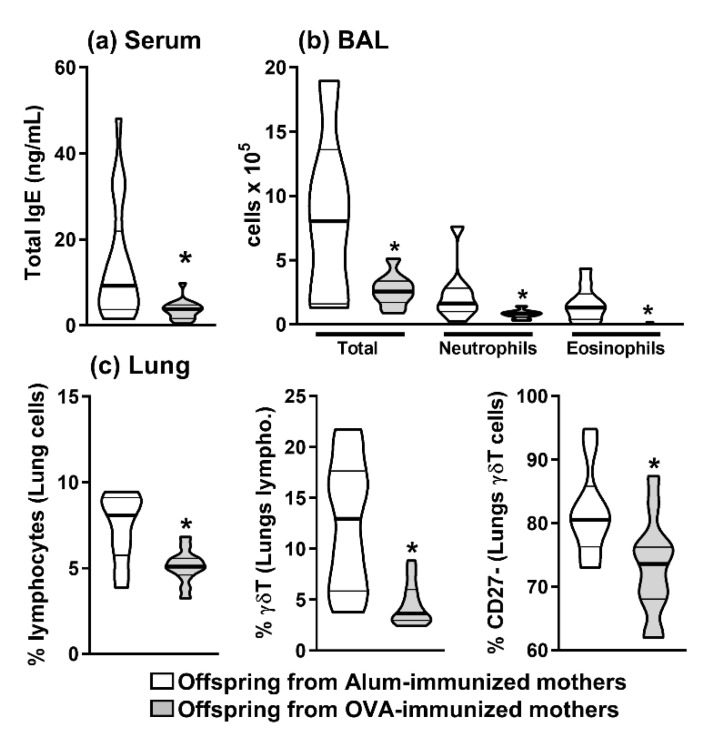
Induction of offspring allergy tolerance mediated by maternal immunization with OVA. Offspring from OVA-immunized (*n* = 10) or Alum-immunized (*n* = 11) mothers were immunized with OVA in the neonatal period. At 20 d.o., offspring total IgE was determined by ELISA (**a**). These groups were subjected to an allergic lung inflammation protocol with OVA, and differential cell counts in BAL (**b**) were evaluated by flow cytometry. The percentage of infiltrated lymphocytes, the frequency of γδT cells, and the frequency of CD27^-^ γδT cells was evaluated in the dissociated lung tissue (**c**) by flow cytometry. The results are illustrated with violin plot (truncated) representing median (bold line) and the quartiles (thin lines). * *p* ≤ 0.05 compared to Alum-immunized offspring.

Using our previously described maternal allergen immunization protocol [6,27,43], we validated the offspring allergic response inhibition immunizing with OVA both offsprings, from OVA-immunized or Alum-immunized mothers, and evaluating some allergy-related biological parameters after pulmonary OVA-challenge. Total serum IgE and allergic pulmonary inflammation in response to OVA, as evaluated by the frequency of neutrophils and eosinophils in BAL analyses, were suppressed in offspring from OVA-immunized mothers (Figure 1a,b). Additionally, we observed that maternal OVA immunization decreased the frequency of infiltrating pulmonary lymphocytes in the offspring lung, and this was mainly mediated by the reduction of γδT cells infiltration (Figure 1c). Because thymic IL-17-producing γδT cells mainly express the CD27^-^ phenotype, we further analyzed the frequency of CD27^-^ γδT cells on the lung, where they could be detected at lower frequencies on offspring from OVA-immunized mothers (Figure 1c). These results validated our maternal OVA-immunization protocol inducing OVA tolerance and decreasing CD27^-^ γδT cells’ frequency. 

**Figure 2:**
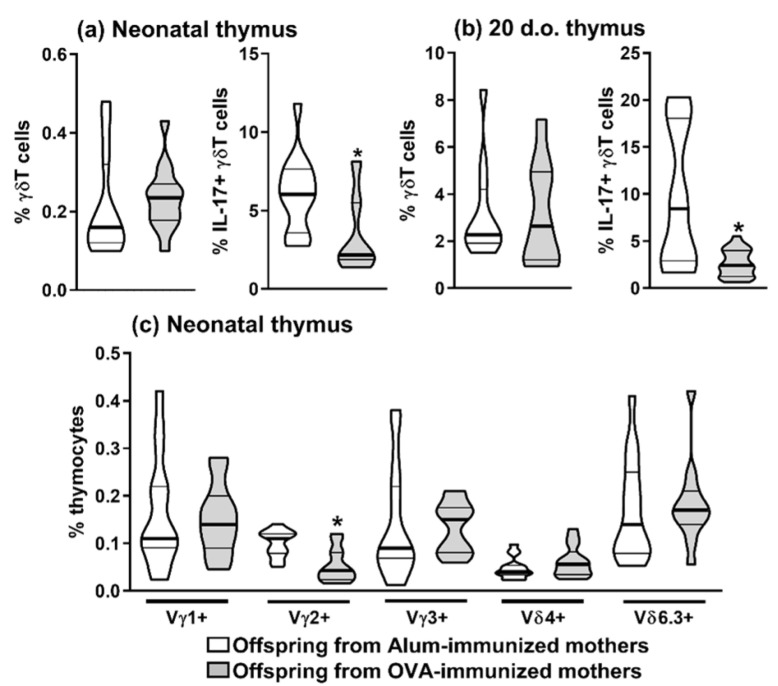
Inhibition of offspring thymic maturation of IL-17-producing and Vγ2^+^ γδT cells mediated by maternal immunization with OVA. Offspring from OVA-immunized (*n* = 9) or Alum-immunized (*n* = 10) mothers were evaluated. Thymic frequency of total and IL-17-producing (IL-17^+^) γδT cells were evaluated by flow cytometry at 3 (**a**) and 20 (**b**) d.o. offspring. Additionally, thymic frequency of Vγ1, Vγ2, Vγ3, Vδ4, and Vδ6.3 γδT cells was evaluated at 3 (**c**) d.o. offspring by flow cytometry. The results are illustrated with violin plot (truncated) representing median (bold line) and the quartiles (thin lines). *p ≤ 0.05 compared to Alum-immunized offspring.

By evaluating the thymuses of offspring at neonatal age, we could observe that maternal OVA immunization could also reduce offspring intrathymic IL-17-producing γδT cell frequency compared to the control group (Figure 2a) and until 20 d.o. offspring thymus (Figure 2b). We also evaluated the expression of the leading, functional-related Vg and Vd chains expressed by thymic γδT cells in the neonatal age, and we could observe that maternal OVA-immunization can reduce the expression of thymic Vg2^+^ γδT cells compared to the control group and without influence on the expression of Vg1, Vg3, Vd4 and Vd6.3 chains (Figure 2c). These results indicated that maternal OVA immunization could exert a pronounced inhibition of offspring thymic IL-17-producing γδT as observed by the reduced frequency of CD27-, IL-17^+^ and Vg2 chain expressed by γδT cells.

### 2.2. Maternal Immunization Could Induce the Expression of IL-17 Inhibition-Related miRNAs on Offspring Thymus

Cleaned data from combined genes that included rRNA, tRNAs, piRNAs, pre_miRNA, mature_miRNA, and snoRNA from both groups were used in downstream analyses. Of the known 4648 small RNA sequences generated from the offspring OVA-immunized and Alum-immunized mothers thymus, 434 were tRNAs, 1181 mmu-miRNA,1431 snRNAs, and 1602 snoRNA. All of the 344 detected novel genes were forms of unknown precursors. A total of 2035 mature mmu-miRs that passed the quality filter were detected in both groups. 

**Figure 3:**
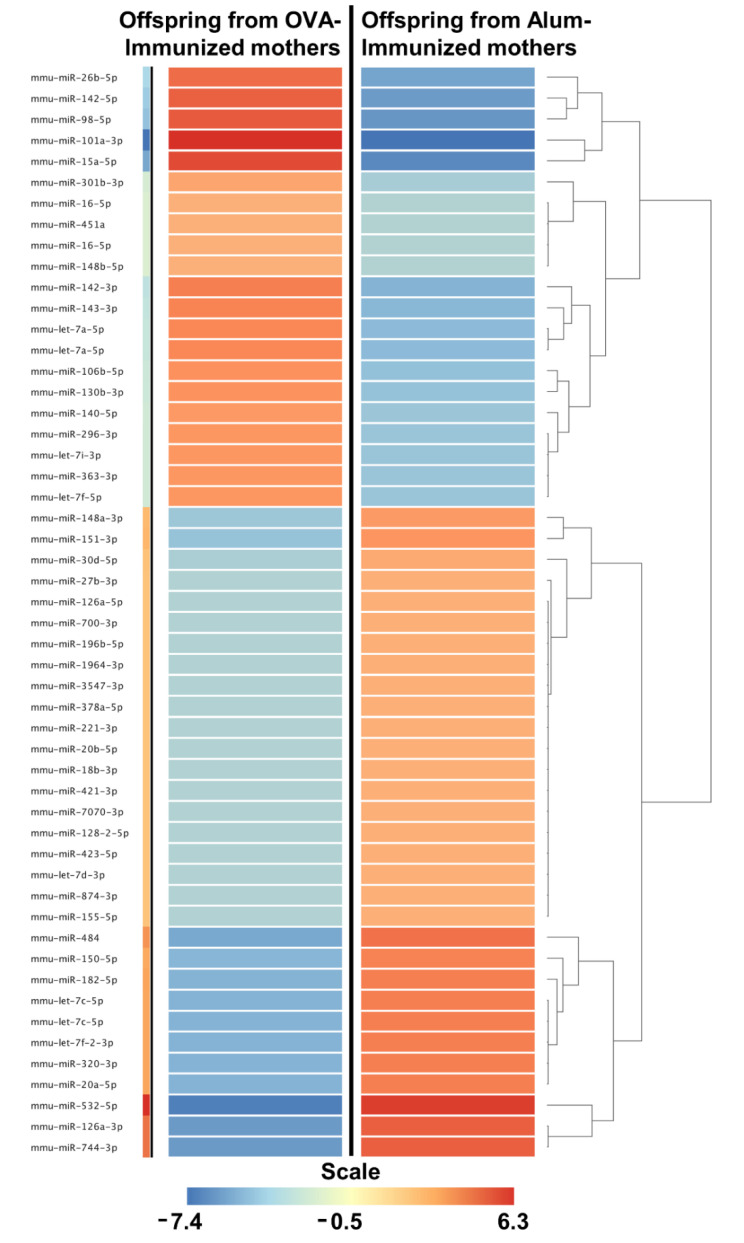
miRNAs that were downregulated by maternal OVA immunization. The thymus of offspring from OVA-immunized (*n* = 10) or Alum-immunized (*n* = 11) mothers was evaluated at three days old. Unsupervised hierarchical clustering demonstrating 52 differentially expressed mature mmu-miRs between offspring OVA-immunized and Alum-immunized mothers. The mmu-miRs clustering tree is displayed to the right, forming two major clusters. The color scale at the bottom indicates the fold change expression levels of mature mmu-miRs across in both samples: red color indicates overexpression and blue underexpression.

**Figure 4:**
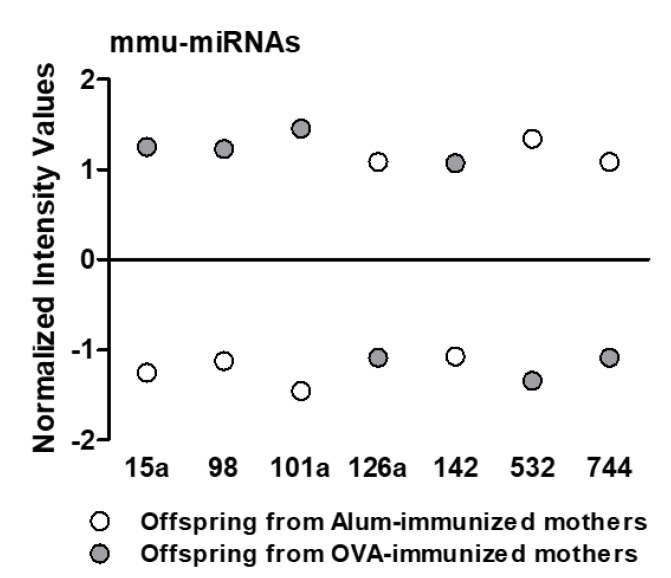
miRNAs that were substantially modulated between groups. The thymus of offspring from OVA-immunized (*n* = 10) or Alum-immunized (*n* = 11) mothers was evaluated at three days old. The dots showed the seven dysregulated miRNAs between offspring from Alum and OVA-immunized mothers. The fold change in these miRNAs was set at cut-off ≥4.

Here, we decided to restrict our expression analysis to the mature mmu-miRs identities between the offspring in both groups because these molecules are the most well-studied non-coding RNAs. To this end, a 2-fold expression difference cut-off based on the mature mmu-miRs profiling in both groups was initially set up. This analysis revealed 52 differentially expressed mature mmu-miRs between offspring from Alum and OVA-immunized mothers and that the number of mmu-miRs that were upregulated in Alum-immunized mothers was larger than the number of mmu-miRs that were downregulated (Figure 3 and Table S1). We considered that the fold change in these miRNAs was not very large, so we mainly set the cut-off criteria to a 4-fold expression difference. In the end, seven differentially expressed miRNAs were found, namely mmu-miR-126a-3p, 101a-3p, 744-3p,142-5p, 15a-5p, 532-5p, and 98-5p. We marked these miRNAs and selected them as the potential mmu-miRs signatures for further analysis. The differential expression of these mature mmu-miRs identities between the offspring in both groups is presented in Figure 4. Because the mmu-miR-142-5p was not found in the miRWalk database, the other six mmu-miRs were used to predict their target genes. The results revealed 17 target genes which were then submitted to enrichment analysis to elucidate their potential function. We found no enriched GO functional annotation and pathway enrichment categories for the target genes given our analysis. 

## 3. Discussion

Our results indicate a reduced frequency of CD27^-^ IL-17-producing γδT cells in an allergy tolerance induction murine model mediated by pre conceptional immunization with OVA, similar to a previously published study [17]. However, here, we also evaluated if maternal immunization’s modulatory effect could influence function-related variable chain expression γδTCRs to consolidate the evidence of functional alterations in offspring γδT cells.

Our results reveal that maternal OVA immunization could reduce the frequency of Vγ2^+^ γδT cells on neonate offspring thymus without influencing other variable chains’ expression (Vg1, Vg3, Vd4, and Vd6.3). The expression of the Vγ2 chain is related to skin-homing [44], CCR6 expression, and IL-17 production in γδT cells from fetal mice [36]. This last study emphasizes the separation of Vγ2^+^ cells from other γδT cells and suggests that the molecular program that specifies IL-17 production in γδT cells may overlap that of TCR chains expression. Our results showed that the reduction of offspring thymic CD27^-^ and IL-17^+^ γδT cells and the unprecedented modulation of the Vγ2 chain in γδT cells lend further support to the previous observations.

Scarce evidence can be found in the literature to elucidate the regulation of γδTCR and IL-17 production by murine thymic γδT cells and the possible influence of maternal immunization in this mechanism is unknown. Therefore, we performed a pilot study to evaluate miRNAs’ expression in the thymus of our experimental groups. This approach paves the way for future studies to elucidate the functional modulation of γδT cells. 

miRNAs constitute a fundamental layer of post-transcriptional regulation, influencing several mammalian genes’ expression [45]. The development of murine γδT cells is not impaired by miRNA ablation [46], but few studies have addressed the role of miRNAs in γδT cell functional differentiation. 

We could also observe that maternal OVA immunization could upregulate the expression of miR-142. This microRNA was identified as a critical and indispensable regulator of thymocyte proliferation by targeting the gene of the cell cycle inhibitor Cyclin-dependent kinase inhibitor 1B (Cdkn1b) [47]. However, the same study suggested that miR-142 possibly exerts a multifaceted role on several target genes that still need to be identified. Our observation suggests that it may also indirectly and specifically impact IL-17-producing γδT cell maturation. 

More recently, a study evaluated by microarray the expression of miRNAs comparing γδT CD27^+^ (IL-17-) with CD27^-^ (IL-17^+^) cells and revealed 35 miRNAs that are differentially expressed between these populations [42]. Interestingly, the same study demonstrated that mmu-miR-142 and mmu-miR-101 overexpression is related to γδT CD27^+^ (IL-17^-^) cells, and we observed in our results that the maternal immunization, which induces the reduction of IL-17-producing γδT cells on offspring, induces the upregulation of mmu-miR-142 and mmu-miR-101 on offspring thymocytes. 

Additionally, a recent study had suggested that mmu-miR-15a deficient mice (miR-15a/16-1 C57BL/6 KO mice) can be related to a lower expression of IL-17 compared to wild type mice, but this effect could be compensated after polyclonal stimulation suggesting that mmu-miR-15a did not affect IL-17 [48]. In contrast, our results indicated the overexpression of mmu-miR-15a on offspring from OVA-immunized mothers, where lower levels of IL-17 production were detected on γδT, this evidence indicates the need for elucidation about the role of mmu-miR-15a on IL-17 production.

Our results also indicated a possible implication to mmu-miR-126 that was downregulated on IL-17-γδT-reduced offspring from OVA-immunized mothers. It was recently described that miR-126 can participate in the regulation of IL-17 production by TCD4 cells in rats [49] and that the downregulation of miR-126 in humans is related to higher levels of IL-17 [50], suggesting that this micro-RNA can influence the production of IL-17. However, there is no evidence about miR-126 regulatory effects on IL-17 in murine models.

Together, evidence found in the literature and our study strongly suggested that maternal OVA immunization can influence offspring’s thymic expression of mmu-miR-15a, mmu-miR-101 mmu-miR-126, and mmu-miR-142, at least as a part of the mechanism of IL-17-producing γδT cells inhibition.

Furthermore, maternal immunization could substantially modulate the expression of some additional miRNAs that are not described as direct regulators of IL-17 production of murine γδT cells. These miRNAs include mmu-miR-98 that were upregulated, and mmu-miR-532 and mmu-miR-744 were downregulated in the same OVA-immunized derived offspring group and others 45 microRNAs that were differentially expressed between evaluated groups. A study suggests mmu-miR-744 as a potent type I interferon inducer [51] and mmu-miR-523 as a pro-inflammatory cytokine attenuation microRNA [52]. These observations may indicate that mmu-miR-744 and mmu-miR-523 could eventually influence the balance between IL-17/IFN-γ production, or the pro-inflammatory profile of γδT cells but, to elucidate the role of these and others microRNAs, further experiments need to be undertaken.

In conclusion, this study demonstrates that the tolerance induction mediated by preconceptional immunization with the allergen OVA can inhibit the maturation of offspring thymic IL-17-producing γδT cells reducing the expression of the function-related Vγ2 chain and inducing the expression of miRNAs that can be related to IL-17 regulation. Thereby, this study features an initial approach linking maternal allergen immunization to epigenetic-mediated modulation of offspring γδT cells.

## 4. Materials and Methods

### 4.1. Immunization

Briefly, C57BL/6 wild-type female mice were immunized subcutaneously with 1500 µg of OVA (Sigma, St Louis, MO, USA) in 6 mg of aluminum hydroxide (Alum; FURP, Sao Paulo, Brazil) or with 6 mg of Alum alone. After 10 and 20 days, these animals were boosted by intraperitoneal route (i.p.) with 1000 µg of OVA in saline or with saline only (Alum-immunized animals). Females were mated 21 days post-immunization. To evaluate offspring IgE responses, pups from Alum- and OVA-immunized mothers were immunized at 3 days old (d.o.) with 150 µg of OVA in 6 mg of Alum, boosted i.p. after 10 days with 100 µg of OVA in saline and bleed at 20 d.o. to obtain serum samples. To evaluate lung inflammation, offspring from both groups were immunized at 25 d.o. with 150 µg of OVA in 6 mg of Alum and boosted (IP) after 10 and 20 days with 100 µg of OVA in saline. These animals were nasally administered with 100 µg of OVA (InvivoGen, San Diego, CA, USA) at 55, 56, 57, 58, and 59 days of age in PBS. Bronchoalveolar lavage (BAL) was obtained at 60 days of age by washing the lungs, and the lungs were surgically removed and subjected to a tissue dissociation protocol. Both methods were previously described [27]. All results were obtained from 9 to 11 pups per group, derived from at least three different mothers, and analyzed in at least three different experiments. All these experiments were approved by the local Animal Ethics Committee (CEUA-IMT: n-000359A- Sao Paulo, SP, Brazil).

### 4.2. Flow Cytometry

Cytometry analyses were performed on the BAL, dissociated lung, and thymus. For BAL cells staining, single-cell suspensions were prepared in FACS buffer (PBS, 1% BSA), and conjugated antibodies (Phycoerythrin—PE, Allophycocyanin—APC, BD Horizon-V450—V450, Peridinin-Chlorophyll-Protein—PercP, or Fluorescein—FITC) recognize murine CD4, CD8, CCR3, MHC-II, CD3, and CD45 (all provided by BD Biosciences, Franklin Lakes, NJ, USA) were used at optimal concentrations. For dissociated lung cells staining, single-cell suspensions were prepared in FACS buffer (PBS, 1% BSA) and conjugated antibodies (PE, APC, V450, or FITC) recognizing murine CD3, γδTCR, CD27 (all provided by BD Biosciences). For thymus cell surface staining, single-cell suspensions were prepared in FACS buffer (PBS, 1% BSA). Conjugated antibodies (PE, APC, BD Horizon-V450, PercP, PercP-Cy5 or FITC) recognizing murine CD3, γδTCR, CD27, Vγ1, Vγ2, Vγ3, Vδ4 and Vδ6.3 (all provided by BD Biosciences).

All antibodies were used at optimal concentrations after the titration experiments. Cell gating strategies were based on specific isotype control values and the fluorochrome minus one (FMO) setting. The strategy to identify cell populations evaluated in this study was the same as previously described [17], except for Variable chains (γ and δ) analyses that are illustrated in Supplementary figures (Figures S1 and S2).

To evaluate intracellular IL-17 production, we adopted a previously standardized protocol for spontaneous cytokine production analyses for intracellular cytokine staining [17,21,27,43]. Briefly, thymocytes were cultured for 24 h at 3 × 10^6^ cells/mL in RPMI (Gibco—ThermoFisher, Waltham, MA, USA) supplemented with 10% heat-inactivated fetal clone (FCS-III, HyClone, Logan, UT, USA) sera without stimulus and in the presence of 10 µg/mL brefeldin A (Sigma, St Louis, MO, USA). Cells were first stained for surface markers, followed by fixation, permeabilization, and intracellular staining with conjugated antibodies that recognize murine IL-17 (BD Biosciences).

For the cell viability analysis, extracellular staining was performed as described above, and the cells were incubated with a Live/Dead (PE-Texas red) fluorescent reagent (ThermoFisher, Waltham, MA, USA). All analyses were performed using viable cells.

Acquisition of 300,000 events per sample was performed in the lymphocyte quadrant (as determined by ratio size/granularity) on an LSRFortessa cytometer (BD Biosciences), and analysis was performed using FlowJo software v10.1 (Becton, Dickinson & Company, Ashland, OR, USA). Statistical analysis was performed with GraphPad Prism v5.0 (GraphPad Software Inc., La Jolla, CA, USA). Data from in vivo studies were taken from 3 to 5 separate experiments with 11 to 22 mice per group. Differences were considered significant at *p* ≤ 0.05, as assessed by the Mann-Whitney U test.

### 4.3. sRNA Sequencing

#### 4.3.1. RNA Extraction

For microRNA sequencing, pools of thymus tissue samples (from 5 to 10 mice) from offspring OVA-immunized or Alum-immunized mothers were collected. Total RNA was extracted using TRIzol (Invitrogen) and purified with RNeasy mini kit (QIAGEN) following the manufacturer’s protocols. sRNAs quantity was measured by a Qubit v2.0 fluorometer (ThermoFisher, Waltham, MA, USA).

#### 4.3.2. sRNA Construction and Sequencing

For each sample in both groups, sRNA libraries were generated by the Small RNA v1.5 sample preparation kit as per the manufacturer’s instructions (Illumina, San Diego, CA, USA) and a previously published protocol [53]. A total library pool of 4 nM was prepared using a MiSeq Reagent Kit v3 150 cycle followed by sequencing on a MiSeq system (Illumina, San Diego, CA, USA). 300-PE run on the MiSeq with a 36-base single-end protocol as previously described [54].

#### 4.3.3. sRNA Data Analysis and Interpretation

After trimming adapter sequences and sequence quality testing, each library’s raw data were aligned to the human reference genome (hg19) using the Strand NGS v3.1 package. Only the miRNA sequences that met the minimum read coverage of ≥5 in each pool were considered for further analyses. Different cut-offs of fold change were used to identify differentially expressed miRNAs. Hierarchal clustering for the significantly differentially regulated miRNAs was plotted using the Strand NGS v3.1 package

#### 4.3.4. Target Genes and Gene Ontology (GO) Analysis

To predict the miRNA-targeted mRNAs, the online miRWalk v3.0 tool (http://mirwalk.umm.uni-heidelberg.de/ accessed on 17 June 2021) was used [54]. After obtaining a list of validated mRNAs genes relative to the seven miRNAs, we scanned these genes and analyzed them for the cellular, molecular, and biological process of GO analysis and Kyoto encyclopedia of genes and genomes (KEGG) pathway.

All sequence data described here are available in the online Zenodo repository https://doi.org/10.5281/zenodo.4657056 (accessed on 17 June 2021).

## Data Availability

The data presented in this study are openly available in the online Zenodo repository at https://doi.org/10.5281/zenodo.4657056 (accessed on 17 June 2021).

## Supplementary Materials

The following are available online at https://www.mdpi.com/article/10.3390/ijms22126633/s1, Figure S1: Illustrative dot plots of the gating strategy used to identify thymic γδT cells in neonatal offspring thymus; Figure S2: Illustrative dot plots of the gating strategy used to identify thymic variable chains expression on neonatal thymic γδT cells. Table S1: thymic small RNA expression on thymus from both offspring groups.
