# Supplementary material for: Preconceptional Immunization Can Modulate Offspring Intrathymic IL-17-Producing γδT Cells with Epigenetic Implications Mediated by microRNAs
Source: Int J Mol Sci. 2021 Jun 21;22(12):6633. doi: 10.3390/ijms22126633 (PMC8234718; doi:10.3390/ijms22126633)
Supplement: Supplementary file 1 [file ijms-22-06633-s001.zip › ijms-1190044-supplementary.pdf]

Title: *Preconceptional immunization can modulate offspring intrathymic IL-17-producing γδT cells: possible epigenetic implications mediated by microRNA*

Authors: Thamires Rodrigues de Sousa, Rodrigo Pessôa, Andrezza Nascimento, Beatriz Oliveira Fagundes, Fábio da Ressureição Sgnotto, Alberto José da Silva Duarte, Sabri Saeed Sanabani and Jefferson Russo Victor.

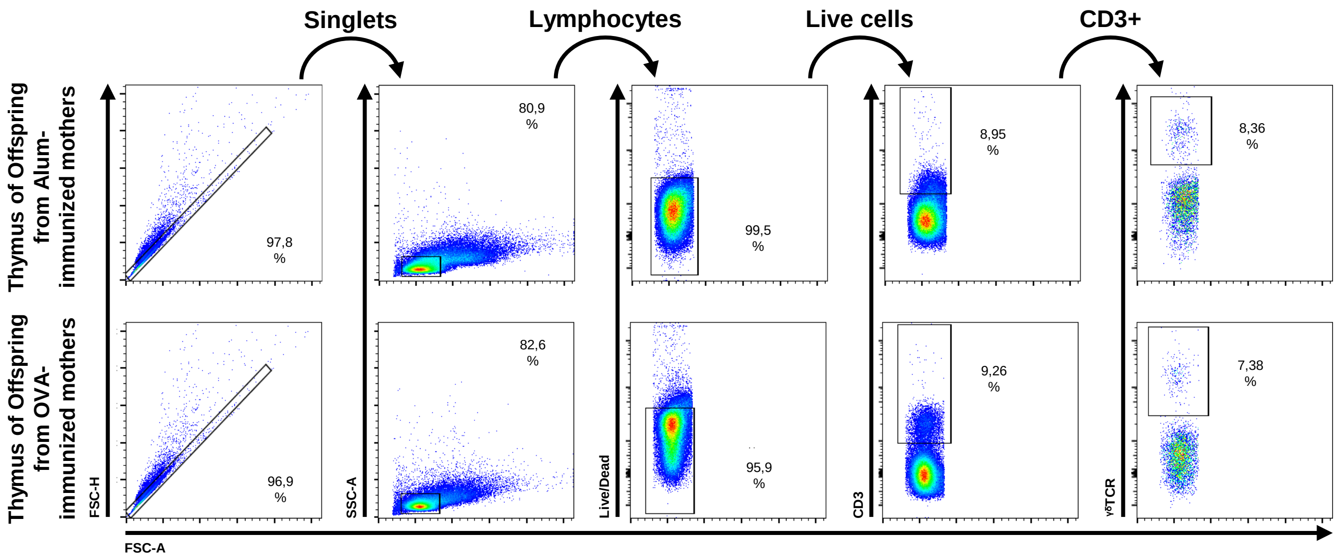

Figure S1: Illustrative dot plots of the gating strategy used to identify thymic γδT cells in neonatal offspring thymus. Each sample was acquired by a consecutive approach using a single-cell gate (determined by FSC-A/FSC-H parameters), a lymphocyte gate (determined by relative SSC-A/FSC-A), a live cell gate (determined by Live/Dead staining), a CD3+ cell gate, and, finally, a γδTCR+ cell gate. This figure illustrates the gating strategy in both experimental groups (Alum-immunized mothers and OVA-immunized mothers).

(a)

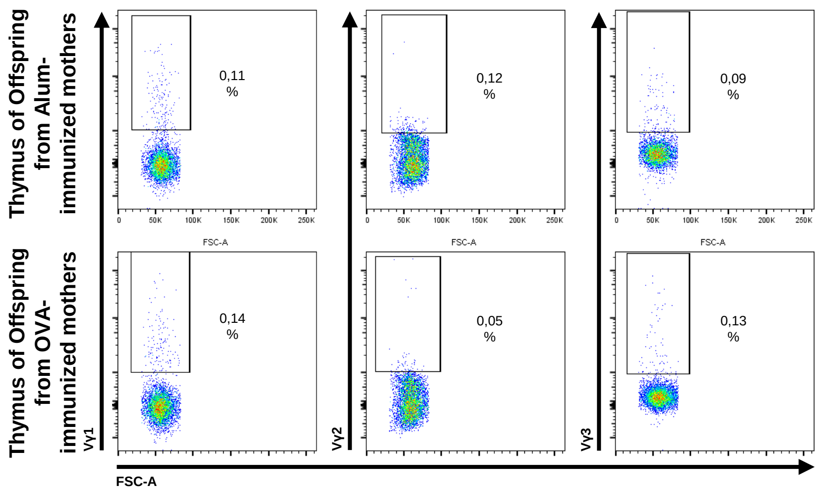

(b)

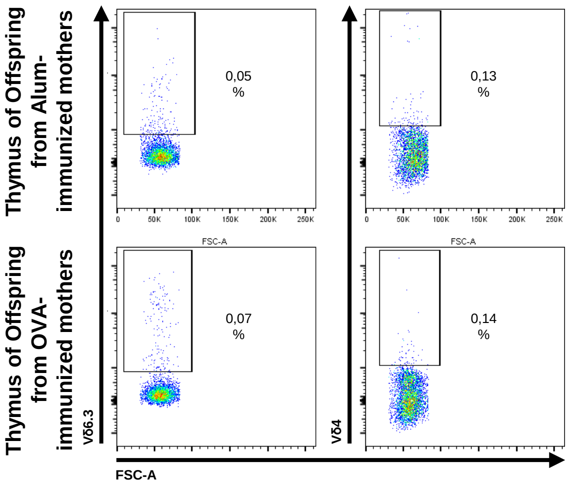

Figure S2: Illustrative dot plots of the gating strategy used to identify thymic variable chains expression on neonatal thymic γδT cells. As demonstrated in figure S1, each sample was acquired by a consecutive approach using a single-cell gate, a lymphocyte gate, a live cell gate, a CD3+ cell gate, and, finally, a γδTCR+ cell gate. These samples were then acquired and gated as Vγ1+, Vγ2+ or Vγ3+ (a), and Vδ6.3+ or Vδ4+ (b). This figure illustrates the gating strategy in both experimental groups (Alum-immunized mothers and OVA-immunized mothers).
